# ZNF384–ZEB1 feedback loop regulates breast cancer metastasis

**DOI:** 10.1186/s10020-022-00541-1

**Published:** 2022-09-13

**Authors:** Qing-Xiang Meng, Ke-Nie Wang, Jun-Hui Li, Hui Zhang, Zhao-Hui Chen, Xue-Jie Zhou, Xu-Chen Cao, Ping Wang, Yue Yu

**Affiliations:** 1 The First Department of Breast Cancer, Tianjin Medical University Cancer Institute and Hospital, National Clinical Research Center for Cancer, Huan-Hu-Xi Road, He-Xi District, Tianjin, 300060 China; 2 Key Laboratory of Breast Cancer Prevention and Therapy, Tianjin Medical University, Ministry of Education, Tianjin, 300060 China; 3 Key Laboratory of Cancer Prevention and Therapy, Tianjin, 300060 China; 4 Tianjin’s Clinical Research Center for Cancer, Tianjin, 300060 China; 5 Department of Radiobiology, Tianjin Medical University Cancer Institute and Hospital, National Clinical Research Center for Cancer, Huan-Hu-Xi Road, He-Xi District, Tianjin, 300060 China

**Keywords:** ZNF384, ZEB1, miR-485, Epithelial to mesenchymal transition, Metastasis, Breast cancer

## Abstract

**Background:**

Breast cancer has become the most frequently diagnosed cancer worldwide. Increasing evidence indicated that zinc finger proteins (ZNFs), the largest family of transcription factors, contribute to cancer development and progression. Although ZNF384 is overexpressed in several types of human cancer, the role of ZNF384 in breast cancer remains unknown. Therefore, our research focused on ZNF384 regulation of the malignant phenotype of breast cancer and the underlying molecular mechanisms.

**Methods:**

CCK-8 and colony formation assays were used to evaluate cell proliferation. Transwell and scratch assays were used to evaluate the cell migration and invasion. Chromatin immunoprecipitation (ChIP)-qPCR and luciferase reporter assays were used to confirm the target relationship between ZNF384 and zinc finger E-box binding homeobox 1 (ZEB1). Xenografts were used to monitor the targets in vivo effects.

**Results:**

We noted that ZNF384 was significantly overexpressed in breast cancer and highlighted the oncogenic mechanism of ZNF384. ZNF384 transactivated ZEB1 expression and induced an epithelial and mesenchymal-like phenotype, resulting in breast cancer metastasis. Furthermore, ZNF384 may be a target of miR-485-5p, and ZEB1 can up-regulate ZNF384 expression by repressing miR-485-5p expression. Together, we unveiled a feedback loop of ZNF384–ZEB1 in breast cancer metastasis.

**Conclusions:**

The findings suggest that ZNF384 can serve as a prognostic factor and a therapeutic target for breast cancer patients.

**Supplementary Information:**

The online version contains supplementary material available at 10.1186/s10020-022-00541-1.

## Background

Breast cancer has surpassed lung cancer in new cases, becoming the most prevalent cancer in 2020 and the fifth leading cause of cancer-related mortality (Sung et al. 2020). Although earlier diagnosis and effective treatment have improved the prognosis of breast cancer patients, metastasis remains to be the leading cause of cancer-related mortality. Therefore, a better understanding of the mechanisms of metastasis in breast cancer will be beneficial to developing effective therapeutic strategies and improving breast cancer therapy outcomes.

Zinc finger proteins (ZNFs), the largest family of transcription factors, are named after the zinc finger motifs of the DNA-binding domain. The family consists of eight groups according to their structure around the zinc fingers, including Cys2His2 (C2H2)-like, Gag knuckle, Treble clef, Zinc ribbon, Zn2/Cys6, TAZ2 domain-like, Zinc binding loops, and metallothionein (Krishna et al*.* 2003). In addition to binding to the specific DNA sequences, ZNFs interact with RNA, lipids, and other proteins, thereby playing a crucial role in regulating cellular metabolism, including cell proliferation, differentiation, apoptosis, and autophagy. ZNFs have an aberrant expression in a range of cancer cells, such as colorectal (Yang et al. 2008), lung (Jen et al. 2016), and breast cancer (Addison et al. 2015). The overexpression of ZNFs is found in tissue-specific functions as regulators of cancer invasion, migration, and metastasis (Brix et al*.* 2020). For instance, ZNFs directly interact with CtIP to promote its recruitment to DNA double-strand breaks during homologous recombination-mediated DNA repair (Chen et al. 2018) or bind to break sites through the internal C2H2 motif and facilitate the formation of repair complexes during non-homologous end-joining, which may become potential targets for overcoming chemoresistance (Singh et al*.* 2021). However, recent studies have identified several ZNFs as tumor suppressors, which are down-regulated in cancer cells. For instance, the overexpression of SORBS2 diminishes metastatic capacity via MTUS1 to establish the microtubule stabilization of renal cancer cells (Lv et al*.* 2020).

The ZNF384 gene is located on the human chromosome 12p13.31 and encodes the transcription factor that contains 577 amino acids with a molecular weight of 63 kDa. Previous studies have demonstrated that ZNF384 promotes the malignant phenotype of numerous tumors by enhancing their proliferation, motility, and invasion abilities (Yan et al*.* 2022), which have been intensively studied in hematologic malignancies, especially in pediatric B-other acute lymphoblastic leukemia. ZNF384 acts as a fusion gene with more than ten family partners, which is closely related to the immunophenotype of aberrant cell surface markers of myeloid lineage (CD13 and CD33) during leukemogenesis (Qian et al*.* 2017; Zaliova et al*.* 2021). Instead of tumorigenesis, researchers have hypothesized that ZNF384 fusions enhance the oncogenic potential of other mutations (Qian et al. 2017), where they revealed no significant signs of a worsening prognosis in several clinical retrospective analyses depending on the fusion partners (Hirabayashi et al*.* 2017; Qin et al*.* 2021). Furthermore, ZNF384 has emerged as an oncogene by either directly increasing cell proliferation by up-regulating Cyclin D1 (He et al. 2019) or by blocking the activity of a tumor inhibitor DNASE1L3, which weakened the invasion and growth of hepatocellular cancer cells (Xiao et al. 2022). However, the role of ZNF384 in breast cancer progression was mentioned only in one study that used the TCGA database to release the association with prognosis, which is still largely unknown, and the mechanisms have yet to be clarified (Wan et al*.* 2019).

Here, we investigated the role of ZNF384 in breast cancer metastasis. We discovered that ZNF384 is overexpressed in breast cancer and is predictive of a poor prognosis in patients with breast cancer. Mechanistically, ZNF384 can transactivate Zinc finger E-box binding homeobox 1 (ZEB1) expression, whereas ZEB1 can up-regulate ZNF384 expression by repression of miR-485-5p. Overall, our study demonstrates a novel mechanism of breast metastasis, suggesting that targeting ZNF384 may be an effective strategy for breast cancer therapy.

## Materials and methods

### Oligonucleotides, plasmid, and transfection

The miR-485-5p mimics/inhibitor, siRNAs targeting ZEB1, and ZNF384 and corresponding controls were purchased from RiboBio (China), the oligonucleotides of which are listed in Additional file 1: Table S1, including the ZEB1 promoter regions (− 986 to + 101, − 700 to + 101, − 300 to + 101, and − 100 to + 101) and the miR-485 promoter region (− 1000 to + 58, − 800 to + 58, and − 500 to + 58). These sequences were inserted into the pGL3-basic vector (Promega, Madison, WI, USA). The sequences that contain predicted miRNA binding sites or corresponding mutants were synthesized and inserted into the psiCHEK2 vector (Promega) for the miRNA target gene luciferase reporter. The mutant constructs were created using a site-directed mutagenesis kit (Transgen, China). Furthermore, we synthesized human ZNF384 and ZEB1 cDNAs and inserted them into the pcDNA3-HA vector.

### Cell culture and transfection

Six breast cancer cell lines (BT549, MDA-MB-231, BT474, MCF7, ZR-75-30, and T47D), one normal breast cell line (MCF10A), and 293FT were acquired from the Cell Bank of the Chinese Academy of Sciences (China). All cells were cultured as previously described (Yu et al*.* 2018; Liu et al*.* 2020). Transient transfections were conducted following the standard protocol provided by FuGENE^®^ HD Transfection Reagent (Promega), with each transfection system containing 2 μg plasmid DNA. Stable transfections were conducted with specific lentiviruses (RiboBio), followed by puromycin (2 μg/mL) selection of infected cells for at least 1 week.

### Reverse transcription-quantitative polymerase chain reaction

mRNA and miRNA were extracted using TRIzol^®^ Reagent (Life Technologies) or mirVana miRNA Isolation kit (Life Technologies). The expression levels of mRNA or miRNA were determined using GoTaq^®^ qPCR Master Mix (Promega) or TaqMan miRNA assay kit (Life Technologies) as previously described (Ji et al*.* 2020). GAPDH or U6 expression was used to normalize the target gene expression. Additional file 1: Table S2 outlines the primer sequences.

### Western blot, immunofluorescence, and immunohistochemistry assays

After the proposed experiments, cells were lysed in ice-cold RIPA buffer, and proteins were resolved by SDS-PAGE, transferred to a PVDF membrane, and then incubated with primary antibodies at 1:1000 dilution. After three washes with 1 × TBST buffer for 60 min, HRP-conjugated secondary antibody incubation at a dilution of 1:5000 was applied. ECL reagent (Millipore, Bedford, MA, USA) was used to visualize protein bands.

For immunofluorescence analysis, cells were seeded on glass coverslips in 24-well plates, washed with PBS, fixed in 4% formaldehyde solution for 30 min, and then permeabilized with 0.2% Triton X-100/PBS for 15 min. Cells were blocked with 2% BSA in PBS for 30 min. Coverslips were incubated with primary antibodies overnight at 4 °C, followed by incubation with FITC-conjugated secondary antibodies for 1 h at 24 °C, and then stained with DAPI. Finally, coverslips were observed under a fluorescence microscope.

For immunohistochemistry analysis, formalin-fixed, paraffin-embedded sections were deparaffinized and rehydrated, and antigen was retrieved by boiling in sodium citrate buffer. The sections were incubated overnight at 4 °C with primary antibodies at 1:100 dilution, exposed to HRP-conjugated secondary antibody at 1:500 dilution, and then covered with 3,3′-diaminobenzidine (DAB). The slides were examined using a light microscope, and images were captured using a microscopy camera. Additional file 1: Table S3 displays the antibodies to the examined proteins.

### Colony formation assay

The colony formation assay was used to evaluate the clonogenic potential of the cells. Five hundred cells were planted in each 6-well plate and cultured in a 37 ℃ incubator. Fresh medium was replaced every three days. After two to three weeks of culturing, the culture medium was discarded and washed with PBS thrice. After fixing with 4% paraformaldehyde for 30 min, the colonies were stained with 2% crystal violet solution, washed with PBS, and dried thoroughly, and then colonies were counted under a microscope.

### Cell proliferation, migration, and invasion assays

CCK-8 was used to evaluate cell proliferation. CCK-8 analysis was performed using TransDetect^®^ Cell Counting Kit (TransGen, China). Each well of a 96-well plate was inoculated with 100 μL cell suspension (2 × 103 cells per well) and cultured in a 37 ℃ incubator. After the culture period, 10 μL of CCK8 solution was added to each well, and the absorbance value was measured at 450 nm using a microplate reader.

Transwell and scratch assays were used to evaluate the cell migration and invasion. Transwell assay investigates the migration and invasion of cells after the upper chamber was coated with Matrigel (BD Bioscience, New Jersey, USA). A total of 5 × 10^4^ cells were seeded in the upper chamber with the FBS-free medium and in the lower chamber with the culture medium containing 20% FBS. After 16–24 h of culture at 37 ℃, the upper cells were eliminated, and the invaded cells were fixed and dyed using the three-step set (Thermo Scientific, Waltham, MA, USA). The invaded cells were photographed using an inverted microscope imaging system (Olympus) to determine cell migration and invasion. For the scratch assay, hairline scratches were produced using a 10 μL plastic pipette tip until the cell density in each well reached 80%. These scratches were photographed every 12 h. The migration trajectories were observed and analyzed.

### Chromatin immunoprecipitation (ChIP)-qPCR and luciferase reporter assays

ChIP analysis was performed according to the protocol of Upstate Biotechnology as previously described (Yu et al. 2018). To calculate the binding to the DNA region analyzed, the enrichment with the specific antibody compared to the isotype control is expressed as % input. The sequences of oligonucleotides used as ChIP primers are listed in Additional file: Table S4.

For luciferase reporter analysis, 293FT cells were seeded in a 24-well plate at 2 × 10^4^ per well and transfected with FuGENE HD Transfection Reagent (Promega) for 48 h with 100 ng of the indicated firefly luciferase reporter plasmid, 100 ng of pcDNA3-ZNF384/ZEB1, and 20 ng of *Renilla* reporter as a normalization control. The luciferase activity was determined by a dual-luciferase reporter assay kit (Promega) according to the manufacturer's protocols.

### Xenograft

For proliferation analysis, Cells (1 × 10^7^) were subcutaneously inoculated in female NSG mice (5 ~ 7 weeks old). The tumor size was examined every 5 days using electron vernier calipers, and the tumor volume was computed using the formula [V = (L × W)^2^/2]. After 35 days, the animals were terminated, and the weight and size of tumor tissue were measured. For metastasis analysis, breast cancer cells (5 × 10^5^) were intravenously injected into NSG mice. Metastatic clone formation was assessed by bioluminescence imaging every seven days via in vivo imaging systems. All animal experiments are approved by our Animal Ethics Committee and meet the animal welfare guidelines.

### Statistical analysis

SPSS 24.0 (IBM, Armonk) was used for data analysis. All measurement data were exhibited as mean ± standard deviation. Statistically significant deficiencies between groups were determined by one-factor analysis of variance (ANOVA) or Student’s t-test. A double-tailed p-value of less than 0.05 denoted statistical significance.

## Results

### ZNF384 is highly expressed in breast cancer and associated with a poor prognosis

**Fig. 1:**
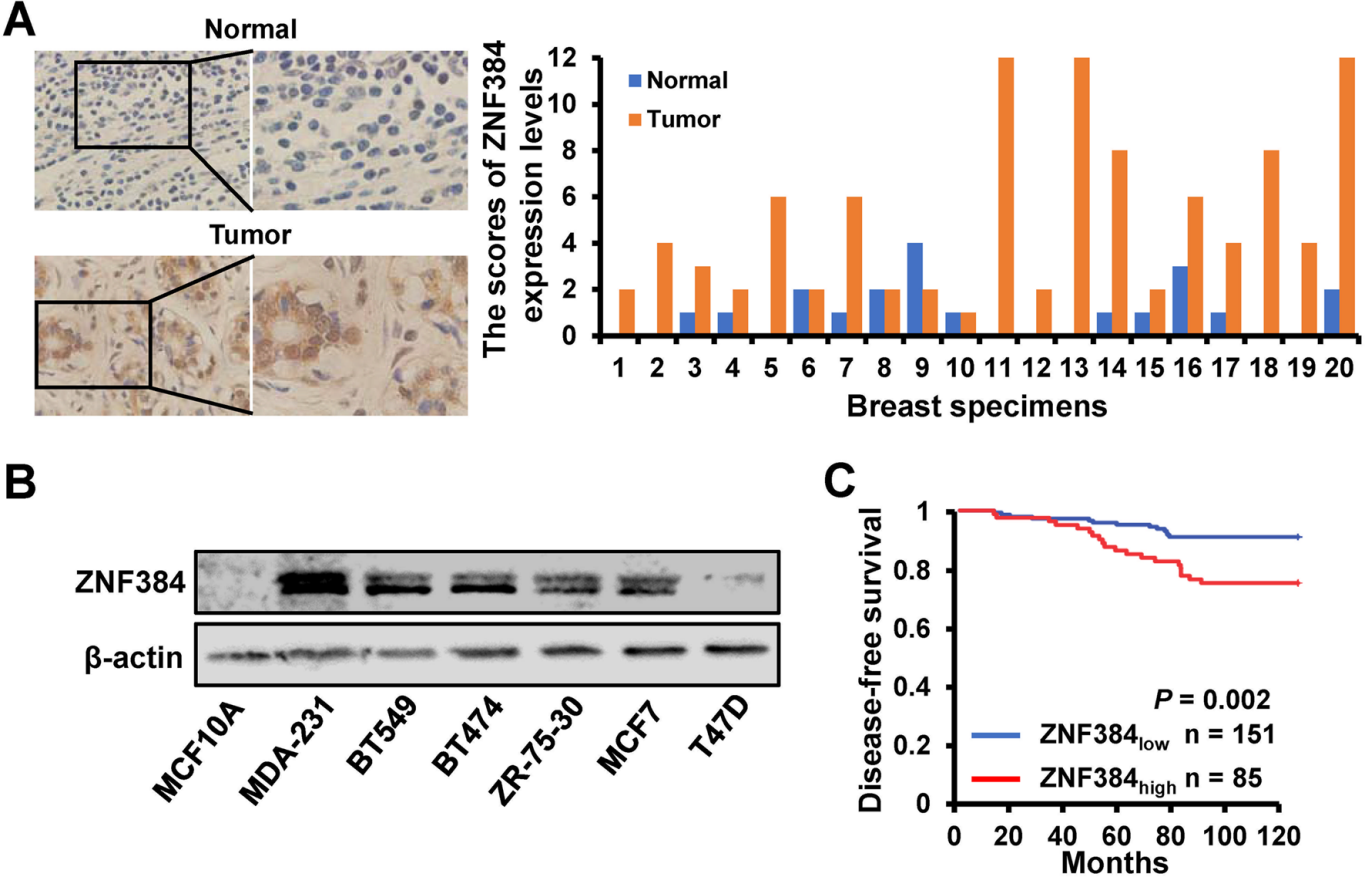
ZNF384 is highly expressed in breast cancer and associated with poor prognosis. **A** ZNF384 expression determined by IHC in 20 cases of primary breast cancer tissues and the paired normal breast tissues. **B** ZNF384 expression in six breast cancer cells and the normal breast cell line MCF10A determined by western blot. **C** The Kaplan–Meier analysis of disease-free survival of patients with different ZNF384 expression levels (n = 236)

To investigate the role of ZNF384 in breast cancer progression, we determined the expression levels of ZNF384 in 20 cases of primary breast cancer tissues and the paired adjacent normal tissues by Immunohistochemistry (IHC). As illustrated in Fig. 1A, ZNF384 is mainly localized in the nucleus and is overexpressed in breast cancer tissues than normal breast tissue. Moreover, ZNF384 expression was up-egulated in six different breast cancer cell lines compared with the normal breast cell line MCF10A by western blot (Fig. 1B). Clinically, patients with high ZNF384 levels exhibited a remarkably worse prognosis than those with low ZNF384 levels (Fig. 1C). Together, these findings indicate that ZNF384 may contribute to breast cancer progression.

### ZNF384 contributes to breast cancer metastasis

**Fig. 2:**
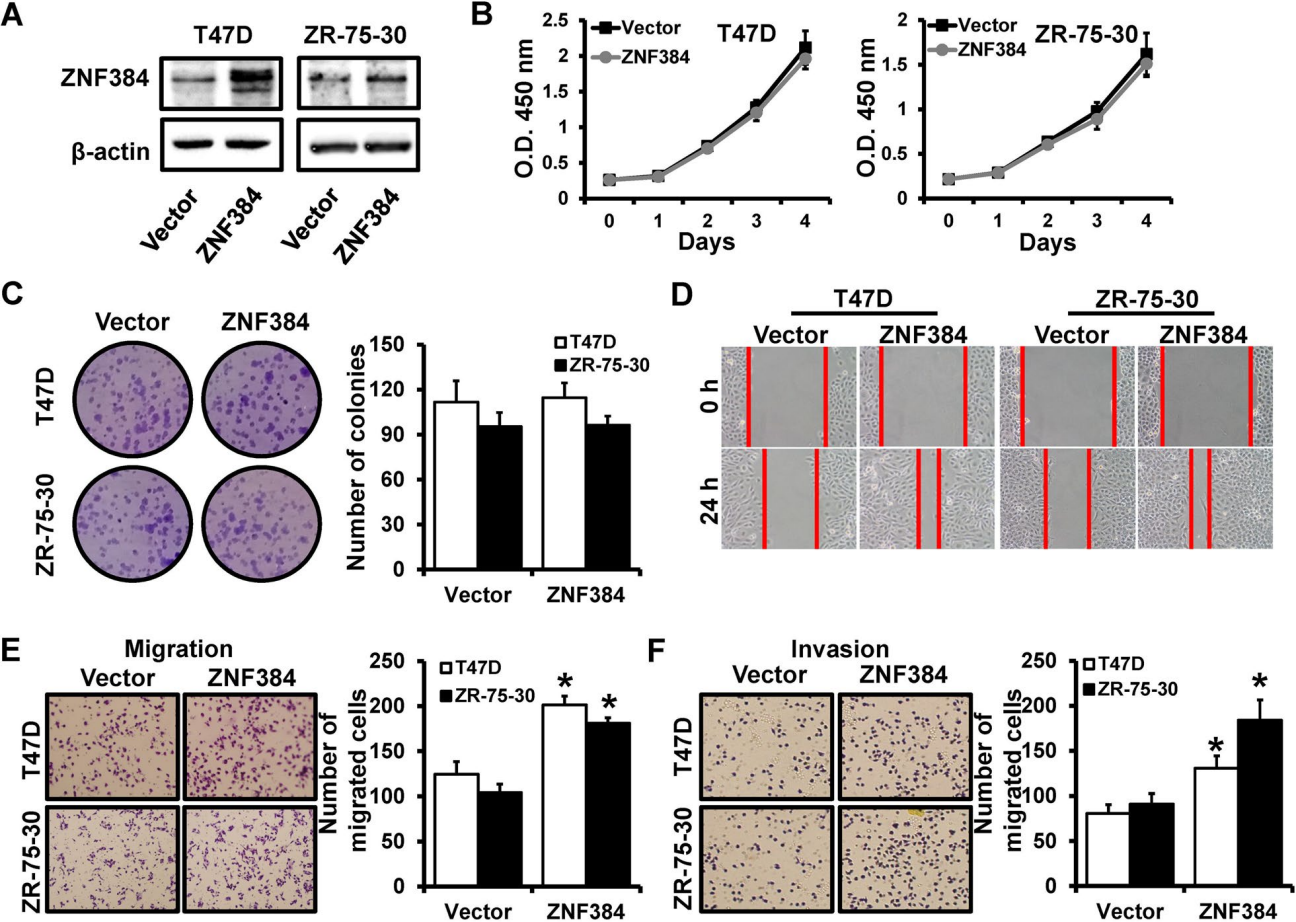
ZNF384 overexpression promotes breast cancer migration and invasion in vitro. **A** ZNF384 expression in T47D and ZR-75-30 cells transfected with a human ZNF384 expression vector, as well as vector control cells determined by western blot. **B** The ability of cell proliferation of cells as in **A** determined by CCK-8. **C** The clonogenic potential of cells as in **A** determined by colony formation assays. **D–F** The ability of cell migration and invasion in cells as in **A** determined by wound-healing (**D**) and transwell (**E**, **F**) assays. *P < 0.05

To further investigate the role of ZNF384 in breast cancer progression, we transfected a human ZNF384 expression vector into T47D and ZR-75-30 cells (Fig. 2A). Forced expression of ZNF384 did not affect the cell proliferation (Fig. 2B) and colony formation (Fig. 2C) in vitro. However, wound-healing (Fig. 2D) and transwell (Fig. 2E, F) assays demonstrated that overexpression of ZNF384 can promote cell migration and invasion. Together, these findings indicate that ZNF384 acts as an oncogene in breast cancer.

**Fig. 3:**
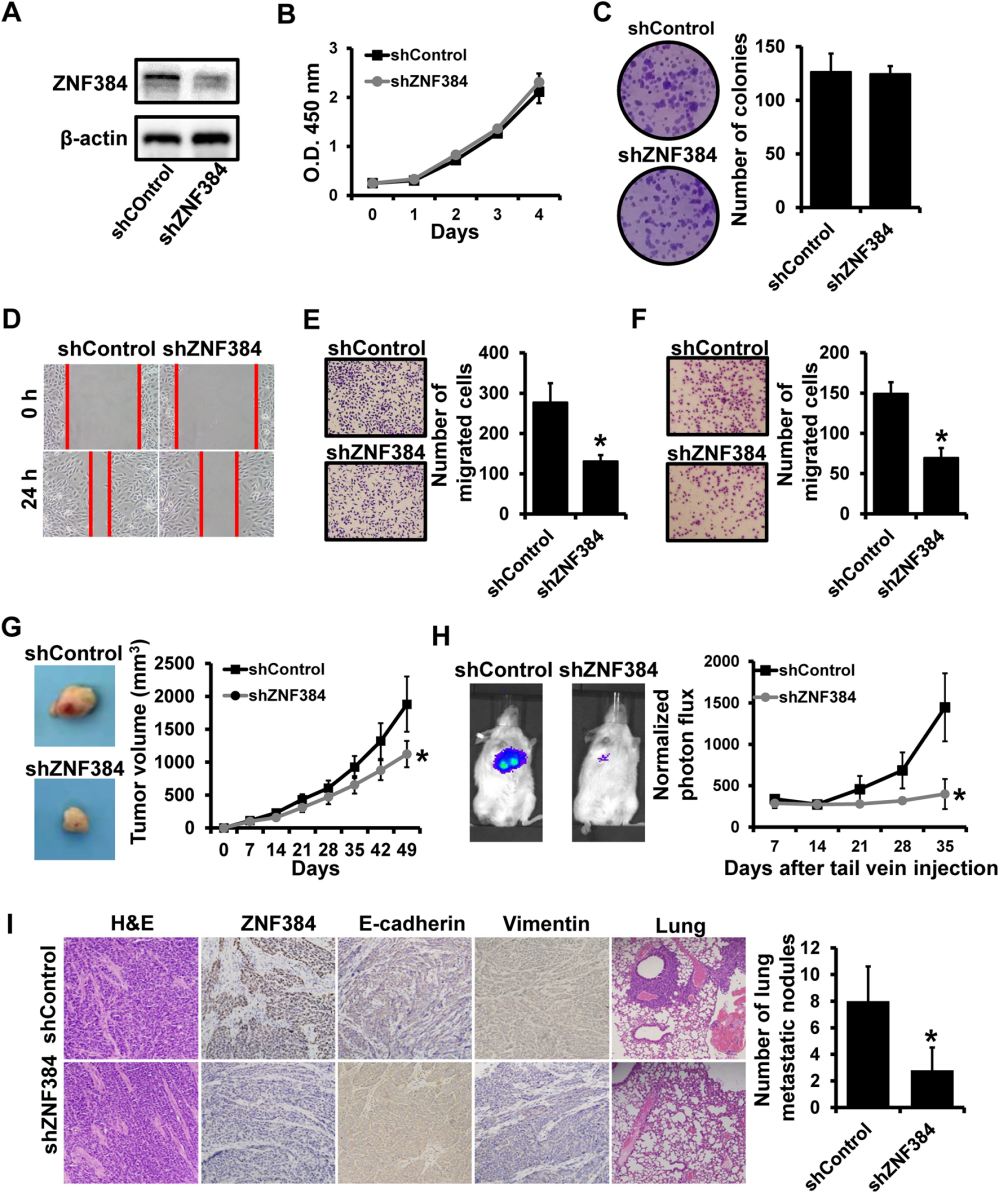
Depletion of ZNF384 expression suppresses breast cancer metastasis.** A** ZNF384 expression in stable ZNF384-depleted cells (shZNF384) and control cells (shControl) determined by western blot. **B** The ability of cell proliferation of cells as in **A** determined by CCK-8. **C** The clonogenic potential of cells as in **A** determined by colony formation assays. **D–F** The ability of cell migration and invasion in cells as in **A** determined by wound-healing (**D**) and transwell (**E**–**F**) assays. **G** Tumor growth curves for xenograft mice injected with cells as in (A) at the indicated times. **H** Bioluminescent imaging of metastasis for xenograft mice after tail vein injection of cells as in **A**. **I** H&E staining or immunohistochemical staining in primary tumors or metastatic nodules in the lung harvested from mice bearing the indicated xenograft tumors. *P < 0.05

We then constructed stable ZNF384-depleted cells (shZNF384) and control cells (shControl) by using the MDA-MB-231-luc cell line via lentiviral infection (Fig. 3A). ZNF384 expression decreased more than 70% in shZNF384 than in control. Similar to the results of gain-of-function assays, the depletion of ZNF384 did not affect the cell proliferation in vitro (Fig. 3B, C), whereas ZNF384 depletion could promote breast cancer migration and invasion in vitro (Fig. 3D–F). To assess the role of ZNF384 in breast cancer progression in vivo, we implanted shZNF384 and shControl cells into mice mammary fat pads and tail vein, respectively. Contrary to in vitro assays, the depletion of ZNF384 in MDA-MB-231-luc cells could reduce tumor growth (Fig. 3G). We then performed bioluminescent analysis to monitor the metastasis in vivo by using a Xenogen IVIS system. We observed that shZNF384 mice were significantly less susceptible to metastatic dissemination than shControl mice (Fig. 3H). H&E staining revealed less cell mitosis in tumors from shZNF384 mice than in tumors from shControl cells (Fig.  3I). Immunohistochemical staining further identified the up-regulation of E-cadherin and the down-regulation of vimentin by nearly 100% in tumors from shZNF384 mice than those from shControl mice (Fig. 3I). The number of metastatic nodules in the lungs of mice injected with shZNF384 cells was significantly lower than that of control mice (Fig. 3I). Together, these findings indicate that ZNF384 contributes to breast cancer metastasis.

### ZNF384 induces an EMT-like phenotype

**Fig. 4:**
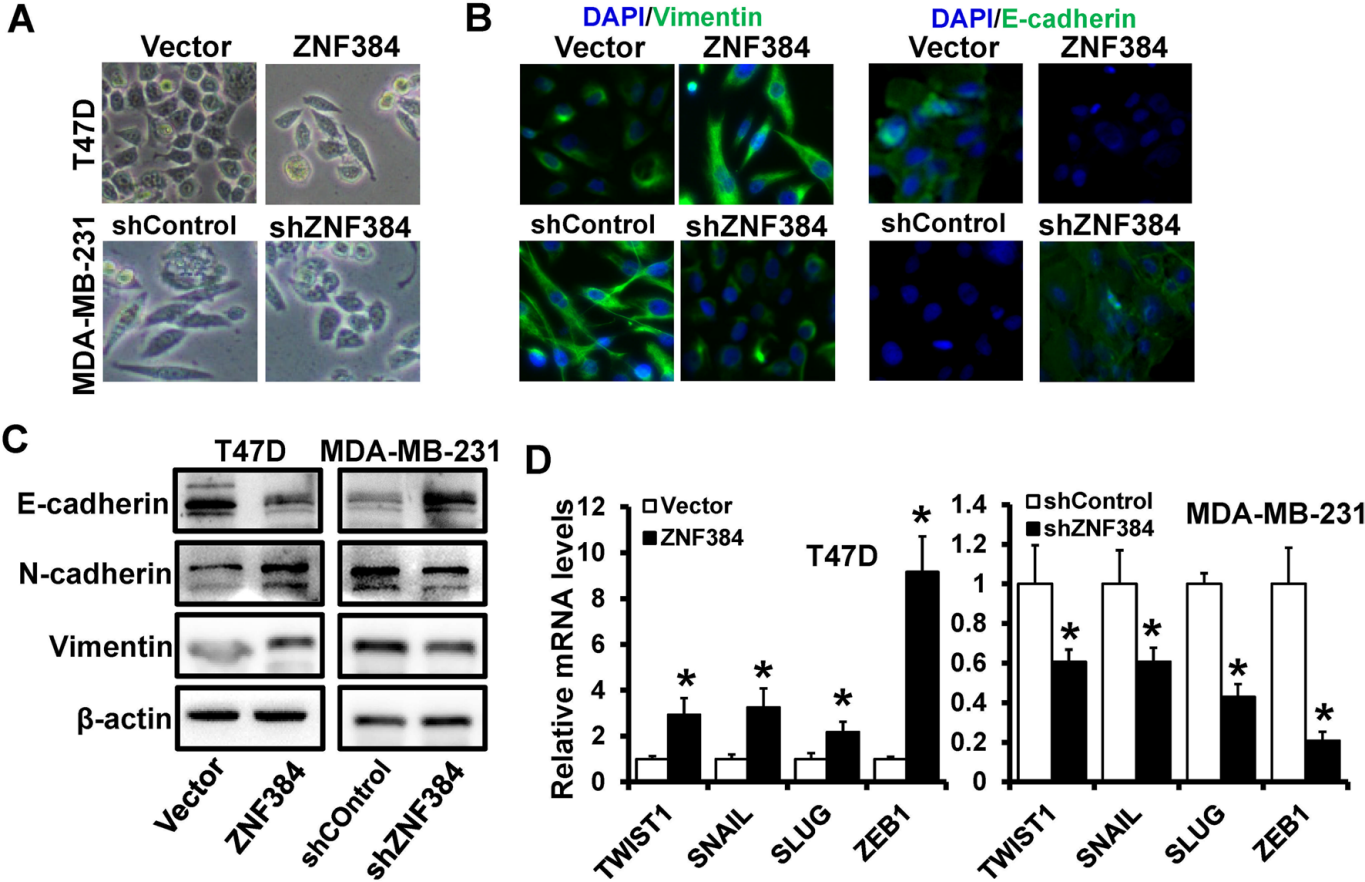
ZNF384 induces an EMT-like phenotype in breast cancer cells.** A** Representative images of morphology in ZNF384-overexpressed T47D cells or ZNF384-depleted MDA-MB-231 cells, as well as control cells. **B, C** The expression of epithelial marker (E-cadherin) and mesenchymal markers (Vimentin and N-cadherin) in cells as in **A** is determined by immunofluorescence (**B**) and western blot (**C**). **D** The expression of EMT-related transcriptional factors in cells as (**A**) determined by RT-qPCR. *P < 0.05

Epithelial-mesenchymal transformation (EMT) is involved in the metastasis process in all types of human cancer; therefore, we evaluated the effect of ZNF384 on EMT in breast cancer. The overexpression of ZNF384 in epithelial T47D resulted in mesenchymal phenotypic conversion, whereas the depletion of ZNF384 in mesenchymal MDA-MB-231 resulted in epithelial phenotypic conversion (Fig. 4A). Consistent with morphologic changes, immunofluorescence staining revealed that ZNF384-overexpressed T47D cells lost the expression of epithelial markers E-cadherin and acquired the expression of mesenchymal marker Vimentin (Fig. 4B). ZNF384-depleted MDA-MB-231 cells maintained the epithelial markers E-cadherin at the cell membrane, whereas the expression of the mesenchymal marker Vimentin decreased than control cells (Fig. 4B). Forced expression of ZNF384 in T47D cells consistently elevated the Vimentin and N-cadherin expression (mesenchymal marker) while decreasing the E-cadherin expression (epithelial marker) than vector cells (Fig. 4C; left). The silencing of ZNF384 expression in MDA-MB-231 cells reduced the expression of Vimentin and N-cadherin while increasing the expression of E-cadherin than shControl cells (Fig. 4C; right). EMT is mainly regulated by a series of transcription factors, including TWIST1, SNAIL, SLUG, and ZEB1. We then determined whether ZNF384 regulates these EMT transcription factors. We observed that the expression of TWIST1, SNAIL, SLUG, and ZEB1 increased in ZNF384-overexpressed T47D cells (Fig. 4D; left), whereas the expression of these transcription factors decreased in ZNF384-depleted MDA-MB-231 cells (Fig. 4D; right) than those of control cells. Together, these findings suggest that ZNF384 induces an EMT-like phenotype in breast cancer.

### ZNF384 promotes breast cancer progression by transactivation of ZEB1 expression

**Fig. 5:**
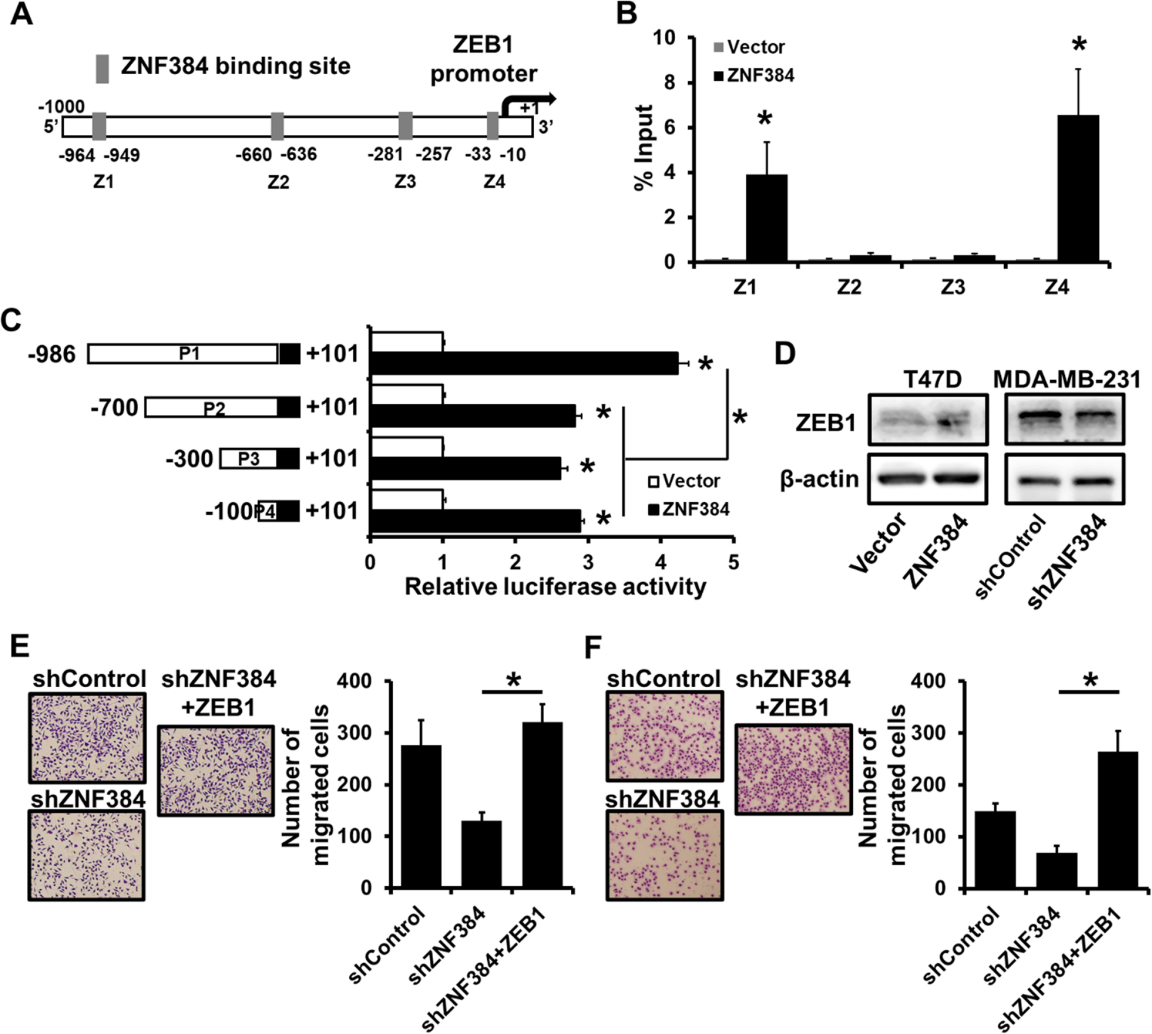
ZNF384 promotes breast cancer progression by transactivation of ZEB1 expression.** A** Four potential ZNF384-binding sites located in ZEB1 promoter region. **B** The interaction between ZNF384 and ZEB1 promoter region in 293FT cells verified by ChIP analysis. **C** The effect of ZNF384 expression on ZEB1 promoter activity determined by dual-luciferase reporter analysis in 293FT cells. A series of fragments containing the predicted ZNF384 binding site is fused upstream of Luc gene. **D** ZEB1 expression determined by western blot in ZNF384-overexpressed T47D cells or ZNF384-depleted MDA-MB-231 cells, as well as control cells. **E–F** The ability of cell migration and invasion in ZNF384-depleted MDA-MB-231 with or without ZEB1 overexpression determined by transwell analysis. *P < 0.05

As previously described, ZNF384 could up-regulate EMT-related transcription factors, and the expression of ZEB1 elevated more than eightfold in ZNF384-transfected T47D cells than in the control group (Fig. 4C). Furthermore, we observed four potential ZNF384-binding sites in the ZEB1 promoter region (− 1000 to + 1; Fig. 5A). The occupancy of ZNF384 on the ZEB1 promoter region was determined using an HA-specific ChIP analysis (Fig. 5B). ZNF384 was able to bind to the ZEB1 promoter region on site 1 (− 964 to − 949) and site 4 (− 33 to − 10; Fig. 5B). We then constructed a series of truncated ZEB1 promoter-reporter to further determine the regions involved in the regulation of ZNF384 by using a dual luciferase reporter assay. We observed that the activation of the ZEB1 promoter was most dominant for the − 986 to + 101 reporters containing two ZNF384 binding sites (Fig. 5C). Moreover, we observed an increased ZEB1expression or a decreased ZEB1 expression in ZNF384-overexpressed T47D cells or ZNF384-depleted MDA-MB-231 cells, respectively (Fig. 5D). Transwell analysis indicated that ZEB1 overexpression rescued the ability of cell migration (Fig. 5E) and invasion (Fig. 5F) in ZNF384-depleted MDA-MB-231 cells. Together, these findings indicate that ZNF384 contributes to breast cancer progression by transactivating ZEB1 expression.

### ZNF384 is a target of miR-485-5p

**Fig. 6:**
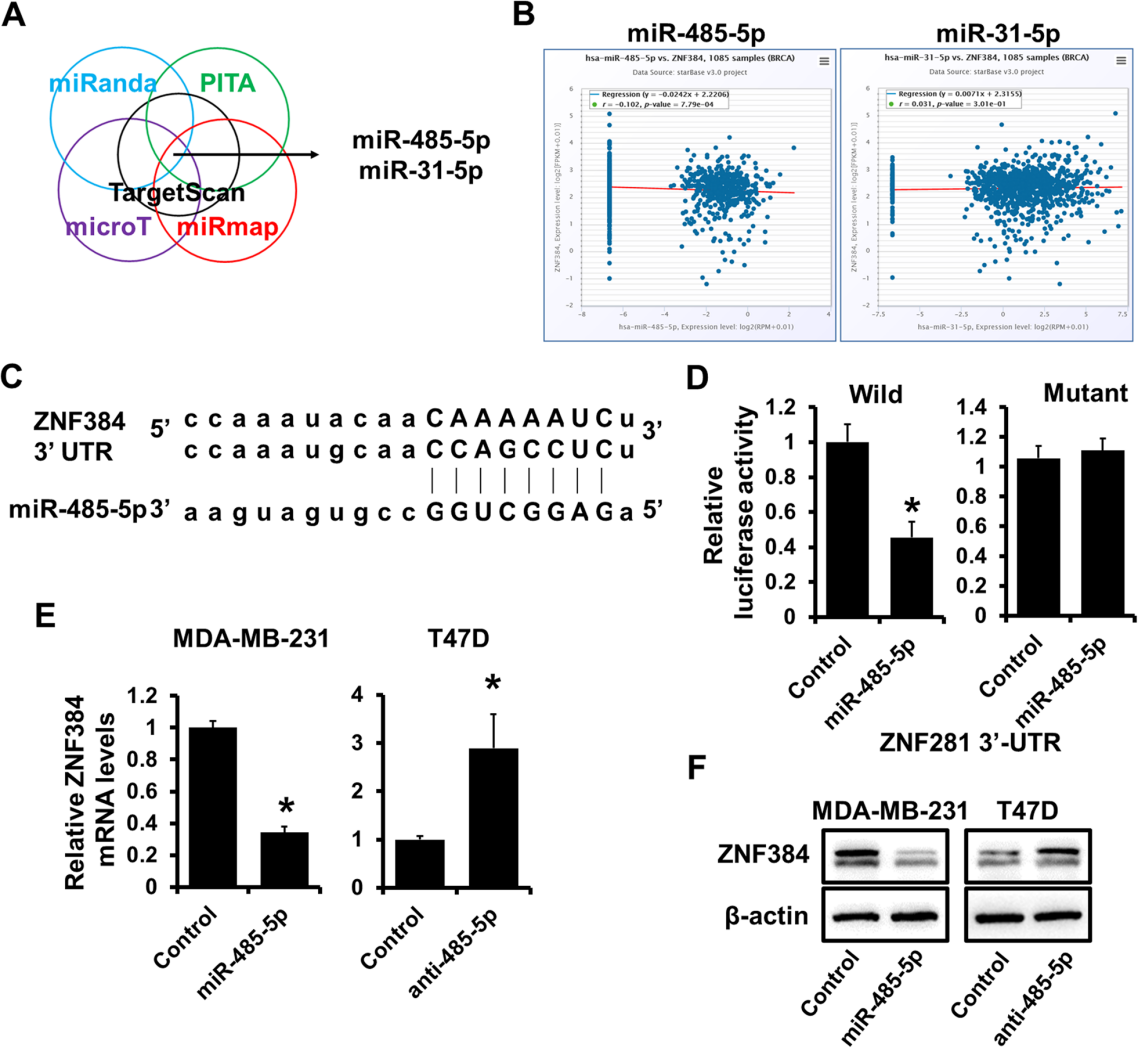
ZNF384 is a target of miR-485-5p. **A** ZNF384 identified as a target of miR-485-5p and miR-31-5p using multiple target-predicting programs. **B** The relationship between ZNF384 and miR-485-5p or miR-31-5p in breast cancer from TCGA data. **C** The predicted binding site of miR-485-5p in ZNF384 3'-UTR. The mut sequence contains a 4-base mutation at the miR-485-5p target seed region. **D** The effect of miR-485-5p expression on wild or mutated ZNF384 3'-UTR activity determined by dual-luciferase reporter analysis in 293FT cells. **E–F** The expression of ZNF384 mRNA levels in miR-485-5p-overexpressed MDA-MB-231 cells or miR-485-5p-depleted T47D cells, as well as control cells, determined by RT-qPCR (**E**) or western blot (**F**). *P < 0.05

Numerous studies have demonstrated that miRNAs can influence the progression of breast cancer by regulating EMT. To elucidate the upstream miRNAs regulating ZNF384, we investigated the upstream miRNAs of ZNF384 and identified two candidate miRNAs using multiple target-predicting programs (Fig. 6A). We then analyzed the relationship between the candidate miRNAs and ZNF384 expression from the TCGA database. We noted that ZNF384 expression was negatively related to the miR-485-5p expression (Fig. 6B). To further confirm this regulation, wild-type or the miR-485-5p binding site mutated ZNF384 3’-UTR was cloned into the psiCHEK2 luciferase reporter (Fig. 6C). Forced expression of miR-485-5p decreased the luciferase activity by approximately 50% compared with the control (Fig. 6D; left), whereas mutation of the predicted binding sites abrogated the miR-485-5p-induced decrease in luciferase reporter activities (Fig. 6D; right). Furthermore, forced expression of miR-485-5p decreased, whereas miR-485-5p depletion up-regulated ZNF384 expression in MDA-MB-231 cells and T47D, respectively (Fig. 6E, F). These findings suggest that ZNF384 is a target of miR-485-5p.

### ZEB1 represses miR-485 transcriptional activity

**Fig. 7:**
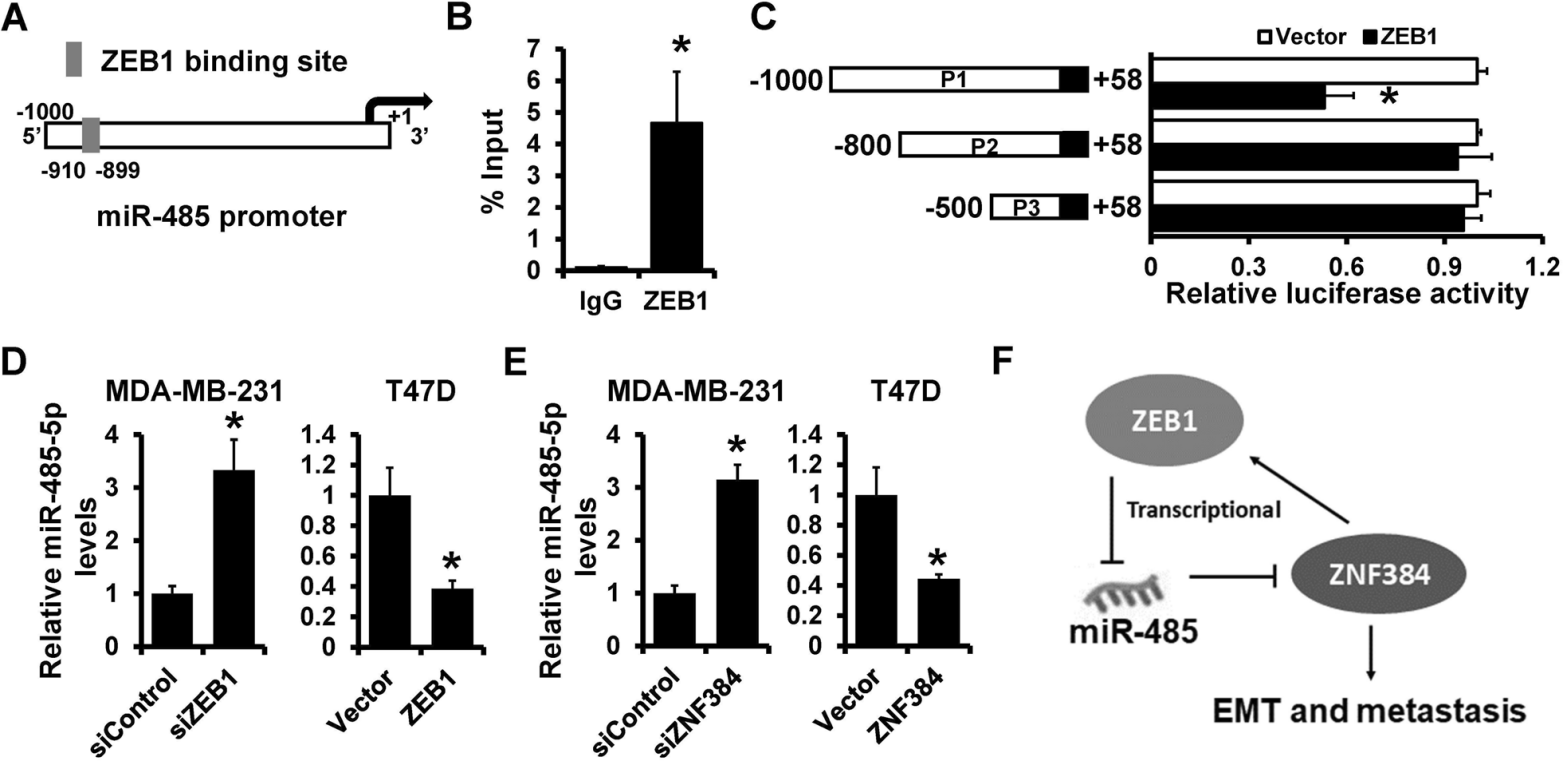
ZEB1 represses miR-485-5p expression in a ZNF384–ZEB1 feedback loop. **A** A potential ZEB1-binding site located in the miR-485 promoter region. **B** The interaction between ZEB1 and miR-485 promoter region in MDA-MB-231 cells verified by ChIP analysis. **C** The effect of ZEB1 expression on miR-485 promoter activity determined by dual-luciferase reporter analysis in 293FT cells. A series of fragments containing the predicted ZEB1 binding site is fused upstream of Luc gene. **D** The expression of miR-485-5p levels in ZEB1-depleted MDA-MB-231 cells or ZEB1-overexpressed T47D cells, as well as control cells, determined by RT-qPCR. **E** The expression of miR-485-5p levels in ZNF384-depleted MDA-MB-231 cells or ZNF384-overexpressed T47D cells, as well as control cells, determined by RT-qPCR. **F** Schematic illustration of ZNF384-miR-485-5p feedback loop in regulating breast cancer EMT and metastasis. *P < 0.05

Our previous studies indicated that ZEB1 could repress multiple miRNAs in breast cancer (Yu et al. 2018). In this view, we sought to determine whether ZEB1 regulates miR-485-5p. Analyses revealed a potential ZEB1 binding site on the miR-485 region (Fig. 7A). ChIP analysis revealed that ZEB1 could bind to the miR-485 promoter in MDA-MB-231 cells (Fig. 7B). We then performed a luciferase reporter assay to determine how ZEB1 expression influenced miR-485 promoter activity. As demonstrated in Fig. 7C, ZEB1 overexpression decreased the wild-type (P1) miR-485 promoter activity, but this effect was nullified in the mutated miR-485 promoter (P2 and P3). Furthermore, ZEB1 knockdown increased the expression of miR-485-5p in MDA-MB-231 cells, whereas ZEB1-overexpression decreased the expression of miR-485-5p in T47D cells than in control cells (Fig. 7D). Furthermore, we observed an increased miR-485-5p expression in ZNF384-depleted MDA-MB-231 and a decreased miR-485-5p expression in ZNF384-overexpressed T47D cells than in control cells (Fig. 7E). These findings suggest that ZEB1 represses the transcriptional activity of miR-485.

## Discussion

Despite advancements in clinical therapy, metastasis is still attributed to almost 90% of cancer-specific deaths (Chaffer and Weinberg 2011). To unveil the role of ZNF384 in cancer progression, we sought to identify the function of this transcription factor and its underlined mechanism. Here, we identified that ZNF384 is an important bridging element in the EMT network, playing a tumorigenic role in metastatic progression in breast cancer cells. Our results uncovered that ZNF384 expression was regulated by a ZNF384/ZEB1/miR-485 feedback loop. ZNF384 directly transactivates ZEB1 expression and induces an EMT-like phenotype and breast cancer metastasis. Furthermore, ZEB1 up-regulates ZNF384 expression by repressing the miR-485-5p expression.

As demonstrated by our results, ZNF384 expression was regulated by the feedback loop involving miR-485 and ZEB1, which is closely related to the poor survival in many cancers (Sakuma et al*.* 2012; He et al. 2019; Gao et al*.* 2021; Xiao et al. 2022). ZEB1 is an activator of EMT by reducing the expression of epithelial markers and a regulator of chemoresistance by reducing double-stranded breaks and promoting DNA damage repair (Zhang et al. 2018). Moreover, increasing evidence points to an association between overexpression of ZEB1 and poor clinic prognosis. Phosphorylation, ubiquitination, and microRNAs effectively regulate ZEB1 expression (Jen and Wang 2016). In our experimental results, ZNF384 manipulated ZEB1 expression, serving a role in oncogenic effects in cancer cells. Furthermore, previous studies have unraveled the negative regulatory relationship between miR-485 and ZEB1, whose exact mechanism of interrelationship remains unknown. Our study deciphered that miR-485 suppressed the ZNF384/ZEB1 axis, inhibiting cancer cell migration in breast cancer (Xie et al*.* 2021). The target ZEB1 inversely regulated the upstream miR-485 expression, forming a feedback loop with ZNF384 as an integral molecule (Sestito et al*.*, 2020; Chen et al*.* 2021).

EMT program supports cancer cells to overcome barriers to normal epithelial tissue and induces invasion and metastasis, thereby accelerating the progression of breast cancer (Valastyan and Weinberg 2011). ZNF384, a recently found transcription factor with the zinc finger domain, exhibits consistently high expression in lung cancer, hepatocellular cancer (He et al. 2019; Xiao et al. 2022), colorectal cancer (Yan et al. 2022), and osteosarcoma (Gao et al. 2021). High expression levels of ZNF384 are of clinical relevance with the worsening prognosis. The underlying mechanism varied depending on the specific context. For instance, ZNF384 is required for DNA repair activity, maintaining cell morphology and genomic integrity, and stabilizing unlimited proliferation during DNA damage (Chen et al. 2018; Singh et al. 2021). In addition, ZNF384 acted as an oncogene by inducing proliferation, apoptosis, and glucose reprogramming by targeting the backup endonuclease DNASE1L3 (Xiao et al. 2022) and elevating cell cycle protein expression (He et al. 2019). Mounting evidence suggested that ZNF384 transactivated multiple downstream oncogenes, including HBO1 and MMP2, highlighting its tumorigenic function in osteosarcoma and colorectal cancer (Sakuma et al. 2012; Gao et al. 2021; Yan et al. 2022). In breast cancer cells, ZNF384 directly transactivated ZEB1 expression by binding to the activation of the promotor. Additionally, the same efficacy can be mediated through other factors and signaling pathways (Bai et al*.* 2014). Activated ZEB1 drove epithelial gene expression and caused a loss of intercellular adhesion, which resulted in cell dissociation (Caramel et al*.* 2018). Activated ZEB1 also elevated mesenchymal factors such as N-cadherin and fibronectin, which are responsible for invasive and migratory capacities (Krebs et al*.* 2017). However, the deletion of ZNF384 did not affect cell proliferation and viability in vitro but still exerted a reduced effect in vivo. We postulated that the cause depended on the microenvironment and other factors that could compensate for the loss, whereas it promoted proliferation in vivo. The regulation of the dynamic process of EMT was complicated, where ZEB1 contributed as a key determinant in a double-negative feedback loop (Bracken et al*.* 2008). ZNF384 shares a homologous zinc finger motif with that of ZEB1. In our experimental findings, force expression of ZNF384 was related to malignancy, whereas depletion of ZNF384 suppressed the colonization capacity of cancer cells. Herein, the probed tumorigenic role of ZNF384 in breast cancer expands the current EMT network, including ZEB1.

microRNAs influence the process of breast cancer in many ways, including the crosstalk between cancer cells and surrounding tissues (Scognamiglio et al*.* 2022), the initiation of EMT, and macrophage reprogramming (Ma et al*.* 2022). In this study, miR-485 directly targeted and reduced ZNF384 and downstream ZEB1 expression, as confirmed by luciferase assays, consistent with previous studies that modulated tumor-suppressive functions by binding to the specific coding sequences downstream (Huang et al*.* 2010; Bai et al. 2014). However, ChIP analysis confirmed the existence of a feedback loop in which high ZEB1 expression transcriptionally inhibits miR-485, thereby allowing cancer cells to maintain their outgrowth capacity. Additionally, low levels of microRNAs were responsible for the chemoresistance of breast cancer through attenuated inhibition of ZEB1 expression at the mRNA and protein levels (Bai et al. 2014). This feedback loop is prevalent throughout cancer progression (Hill et al*.* 2013). Collectively, our results explored the critical oncogenic function of ZNF384 and its regulation by the feedback loop, which can be a potential therapeutic target to inhibit the proliferation and metastasis of cancer cells, as well as re-sensitize them to chemotherapy.

## Conclusion

In conclusion, we unveiled a feedback loop of ZNF384–ZEB1 in breast cancer metastasis (Fig. 7F). The dysregulation of this feedback loop contributes to breast cancer metastasis by inducting an EMT-like phenotype. We propose that ZNF384 can serve as a prognostic factor and target patients with breast cancer. Nevertheless, a large sample of clinical data is required to determine the clinical applications of ZNF384.

## Supplementary Information

**Additional file 1: Table S1. **Oligonucleotides of miRNAs and siRNAs. **Table S2**. Oligonucleotides used for RT-qPCR. **Table S3**. Antibodies used for study. **Table S4**. Oligonucleotides used for ChIP and methylation specific PCR.

## Data Availability

The datasets used and/or analysed during the current study are available from the corresponding author on reasonable request.

## Abbreviations

C2H2: Cys2His2
ChIP: Chromatin immunoprecipitation
DNASE1L3: Deoxyribonuclease 1 like 3
EMT: Epithelial-mesenchymal transformation
IHC: Immunohistochemistry
ZEB1: Zinc finger E-box binding homeobox 1
ZNFs: Zinc finger proteins
