# Supplementary material for: ZNF384–ZEB1 feedback loop regulates breast cancer metastasis
Source: Mol Med. 2022 Sep 13;28:111. doi: 10.1186/s10020-022-00541-1 (PMC9469556; doi:10.1186/s10020-022-00541-1)
Supplement: Supplementary file 1 — Additional file 1: Table S1. Oligonucleotides of miRNAs and siRNAs. Table S2. Oligonucleotides used for RT-qPCR. Table S3. Antibodies used for study. Table S4. Oligonucleotides used for ChIP and methylation specific PCR. [file 10020_2022_541_MOESM1_ESM.pdf]

Table S1. Oligonucleotides of miRNAs and siRNAs

mimic control	UGUACCAAUUUCCAGUGGAGAU
inhibitor control	AUGGUGUUAUCAAGUGUAACAG
miR-485-5p mimics	UAUGCAUUGUAUUUUUAGGUCC
miR-485-5p inhibitor	AUACGUAACAUAAAAAUCCAGG
shZNF384 sense	ccggCGGCAACACAACAAAGATAAACTCGAGTTTATCTTTGTTGTGTTGCCGTTTTTg
shZNF384 anti-sense	aattcAAAAACGGCAACACAACAAAGATAAACTCGAGTTTATCTTTGTTGTGTTGCCG
siZEB1	GCUGUUGUUCUGCCAACAGTT

Table S2. Oligonucleotides used for RT-qPCR

Name	Sequence (5' to 3')
miR-485-5p	CCAAGCTTCACCCATTCCTAACAGGAC
U6	CTCGCTTCGGCAGCACA
ZNF384 up<br>ZNF384 low	GTCTCAGGTCAGATCGAGAACA<br>ACTCTGTGTCCATACTGATGCC
TWIST1 up<br>TWIST1 low	GCAAGAAGTCGAGCGAAGAT<br>GCTCTGCAGCTCCTCGAA
SNAIL up<br>SNAIL low	GTCCGTCTGCCGCACCTGAG<br>ACACGGCGGTCCCTACAGC
SLUG up<br>SLUG low	TTCGGACCCACACATTACCT<br>TTGGAGCAGTTTTTGCACTG
ZEB1 up<br>ZEB1 low	TCAAAAGGAAGTCAATGGACAA<br>GTGCAGGAGGGACCTCTTTA
GAPDH up<br>GAPDH low	CAAGGTCATCCATGACAACTTTG<br>GTCCACCACCCTGTTGCTGTAG

Table S3. Antibodies used for study

Name	Source	Catalog
ZNF384	Abcam	ab176689
β-Actin	Immunoway	YM3028

E-cadherin	Immunoway	YT1454
Vimentin	Immunoway	YT4879
N-cadherin	Immunoway	YT2988
ZEB1	Abcam	ab180905

Table S4. Oligonucleotides used for ChIP and methylation specific PCR

**Name**	**Sequence (5' to 3')**
ZNF384 site 1 up<br>ZNF384 site 1 low	CGAGTGGGGTCCACGTTT<br>CATTATCCGGCTTCAGCG
ZNF384 site 2 up<br>ZNF384 site 2 low	CGCTGAAGCCGGATAATG<br>TTCAGGTCTCGATCCCTC
ZNF384 site 3 up<br>ZNF384 site 3 low	GAGAGACGTTGTAAGTTG<br>GTTGCCGCCCATTATTAT
ZNF384 site 4 up<br>ZNF384 site 4 low	GCCGCAGCCCAGGCTATA<br>CGCGTTTCGCATCCCAGA
ZEB1 site up<br>ZEB1 site low	GGCAGCTTCAGGAAAGAT<br>TCTTCTGGAAGTCGGCCT
